# A novel virtual-reality-based route-learning test suite: Assessing the effects of cognitive aging on navigation

**DOI:** 10.3758/s13428-019-01264-8

**Published:** 2019-06-24

**Authors:** Jan M. Wiener, Denise Carroll, Stacey Moeller, Iram Bibi, Dima Ivanova, Peter Allen, Thomas Wolbers

**Affiliations:** 1 Department of Psychology, Ageing and Dementia Research Centre, Bournemouth University, Poole, UK; 2 Mesulam Center for Cognitive Neurology and Alzheimer’s Disease, Northwestern University, Chicago, IL USA; 3 Department of Creative Technology, Bournemouth University, Poole, UK; 4 German Centre for Neurodegenerative Diseases, Magdeburg, Germany

**Keywords:** Navigation, Route learning, Navigation test, Cognitive aging

## Abstract

Most research groups studying human navigational behavior with virtual environment (VE) technology develop their own tasks and protocols. This makes it difficult to compare results between groups and to create normative data sets for any specific navigational task. Such norms, however, are prerequisites for the use of navigation assessments as diagnostic tools—for example, to support the early and differential diagnosis of atypical aging. Here we start addressing these problems by presenting and evaluating a new navigation test suite that we make freely available to other researchers (https://osf.io/mx52y/). Specifically, we designed three navigational tasks, which are adaptations of earlier published tasks used to study the effects of typical and atypical aging on navigation: a route-repetition task that can be solved using egocentric navigation strategies, and route-retracing and directional-approach tasks that both require allocentric spatial processing. Despite introducing a number of changes to the original tasks to make them look more realistic and ecologically valid, and therefore easy to explain to people unfamiliar with a VE or who have cognitive impairments, we replicated the findings from the original studies. Specifically, we found general age-related declines in navigation performance and additional specific difficulties in tasks that required allocentric processes. These findings demonstrate that our new tasks have task demands similar to those of the original tasks, and are thus suited to be used more widely.

Spatial navigation is a fundamental cognitive ability that is important for mobility and independence. However, a growing number of experimental studies have demonstrated age-related declines in a variety of orientation and navigational tasks, including route learning and repetition (Head & Isom, 2010; Zhong & Moffat, 2016), route retracing (Wiener, Kmecova, & de Condappa, 2012), cognitive mapping (Moffat & Resnick, 2002), and wayfinding (Iaria, Palermo, Committeri, & Barton, 2009). Unfortunately, the rise of, and relatively easy access to, virtual environment technology has led most research groups to design and develop their own navigational tasks and protocols, which makes it difficult to compare findings across studies and research groups. As a consequence, normative data for the most popular or frequent navigational tasks, such as route learning (e.g., Allison & Head, 2017; Head & Isom, 2010; Moffat, Zonderman, & Resnick, 2001), landmark sequence test (e.g., Taillade, N’Kaoua, & Sauzéon, 2016; Taillade et al., 2013), landmark recognition test (e.g., Zhong & Kozhevnikov, 2016; Zhong & Moffat, 2016), and cognitive mapping or landmark placement (e.g., Iaria et al., 2009; Liu, Levy, Barton, & Iaria, 2011), are missing at present. Here we begin to address these problems by developing and evaluating three different route-learning tasks (1) that are based on established and published earlier navigation protocols (Head & Isom, 2010; Waller & Lippa, 2007; Wiener, de Condappa, Harris, & Wolbers, 2013; Wiener et al., 2012), (2) that are easy to adapt to address specific research questions, (3) that we make freely available to other research groups (https://osf.io/mx52y/), and (4) that may be of broader interest to researchers engaged in clinical research with various populations.

Declines in spatial orientation and navigation abilities in both typical and atypical aging have now been well documented (for a recent review, see Lester, Moffat, Wiener, Barnes, & Wolbers, 2017). Successful navigation is based on different navigation strategies and mechanisms, which decline at different rates. In healthy older adults, egocentric navigation strategies, also often referred to as route learning or response strategies, are typically preserved for longer than allocentric strategies, which require the processing and encoding of the spatial relationship between landmarks and/or places (Moffat, 2009). In addition, spatial disorientation is one of the earliest signs of Alzheimer’s disease (AD; Pai & Jacobs, 2004; Serino, Morganti, Di Stefano, & Riva, 2015). For example, middle-aged adults at a high risk of developing AD show poorer spatial abilities (Ritchie et al., 2017) and compromised spatial computations in the entorhinal cortex (Kunz et al., 2015), and spatial memory tasks predict conversion from mild cognitive impairment to AD (Wood, Moodley, Lever, Minati, & Chan, 2016). A likely explanation for the sensitivity of spatial tasks and navigational tasks for atypical aging is that brain areas involved in navigation, in particular the entorhinal cortex and the precuneus, show presymptomatic AD-related pathology (Braak & Del Tredici, 2015; Weston et al., 2016). Assessment of navigation abilities thus has the potential to become a powerful diagnostic tool to support the early and differential diagnosis of atypical aging. However, to achieve this goal, normative data for a series of established navigational tasks, which rely on different navigation mechanisms, are critically needed.

Here, we designed three different navigational tasks based on tasks used in earlier aging studies. The first task is a route repetition task, which belongs to the most frequent human navigational tasks. Route knowledge is typically conceptualized as a series of stimulus–response associations in which places or landmarks become associated with movement directions (e.g., “Turn left at gas station”; Waller & Lippa, 2007). In the absence of landmarks, routes can be remembered simply as a sequence of movement instructions (e.g., left, right, straight, right, left; Waller & Lippa, 2007). Both of these strategies are often referred to as egocentric response strategies as they rely on spatial information encoded in an egocentric reference frame (Wolbers & Wiener, 2014). Route navigation has been suggested to be supported by striatal substructures, in particular the caudate nucleus (Hartley, Maguire, Spiers, & Burgess, 2003; Voermans et al., 2004). Even though older adults can show a spontaneous preference for response-based strategies (Rodgers, Sindone, & Moffat, 2012), which has been related to a shift from hippocampal to caudate processing (Konishi et al., 2013; Lester et al., 2017; Zhong & Moffat, 2018, for recent reviews), they can also experience difficulties or challenges with route learning abilities (Head & Isom, 2010). However, age-related differences in route knowledge—such as knowledge about the specific associations between landmarks and directions—can be ameliorated if both age groups are trained until they have successfully learned the route, which takes longer in the older age group (O’Malley, Innes, & Wiener, 2018).

The second task is a route retracing task. In contrast to route repetition, route retracing refers to navigating a route from the end point back to the start point. In route retracing, decision points are approached from a viewpoint different to that experienced during encoding. Simple S–R associations therefore do not support route retracing. Rather, route retracing requires knowledge about the spatial relationship between the direction from which a decision point is approached and the direction in which the route proceeded. Such knowledge contains information about the spatial relationships between locations or landmarks and has been widely referred to as allocentric (Gramann, 2013; Wolbers & Wiener, 2014; Zhong & Kozhevnikov, 2016). To our knowledge, only a few studies have addressed the effects of cognitive aging on route repetition and route retracing (Allison & Head, 2017; Wiener et al., 2012). Although younger participants outperformed the older age group in both route repetition and route retracing, the performance differences were particularly pronounced in retracing, and in contrast to the repetition condition, older adults did not exhibit significant performance improvement in the retracing condition over the course of the experiment.

The third task, the directional-approach task, is an adaptation of a task developed by Wiener et al. (2013). In this task, participants had to recall from which street they originally approached an intersection, when approaching it from a viewpoint they had not experienced before. Solving this task requires participants to encode the configuration of landmarks at the intersection in relation to the direction from which this intersection was approached originally. In line with the aforementioned conceptualization, performing this task requires an engagement of allocentric representations. Younger participants’ performance increased over the course of the experiment, suggesting that they adopted an allocentric strategy, that is when solving the task they relied on an allocentric reference frame to encode spatial information. Older participants’ performance, in contrast, did not increase over the course of the experiment, suggesting that they encountered difficulties in adopting an allocentric strategy.

The primary aim of the present study was to introduce and evaluate a new virtual environment navigation test suite, which builds on earlier research. When designing the new tasks, we aimed to ensure that the environments looked realistic, and that the tasks were ecologically valid and easy to explain. These design considerations are important when working with older adults who are not used to virtual environments and with people with cognitive impairments to ensure that they easily understand the tasks. Despite adapting the tasks and using different environments (stimulus material), we aimed to keep the task demands identical to those of earlier studies and thus expected involvement of the same cognitive–behavioral processes and mechanisms that are evoked by earlier tasks (Wiener et al., 2013; Wiener et al., 2012). We therefore expected to replicate earlier findings: Specifically, we expected that our younger participant group would outperform our older participant group in all three tasks (Head & Isom, 2010; Wiener et al., 2013; Wiener et al., 2012), we expected route repetition performance to be higher than route retracing performance, and we expected older adults to show impaired learning in the route-retracing task, as compared to younger participants (Wiener et al., 2012). We further predicted performance in the directional-approach task to rely on the amount of misalignment between the encoding and the test perspective (de Condappa & Wiener, 2016) and our older age group to be more strongly affected by larger misalignments than our younger age group (Watanabe, 2011).

In contrast to earlier studies, we tested our participants on all three tasks, which allowed us to relate performance between tasks. We expected navigational tasks that share more cognitive mechanisms or strategies to be more strongly correlated than tasks that share fewer cognitive mechanisms or strategies. Specifically, it has been suggested that both the route-retracing and directional-approach tasks require allocentric processing (de Condappa & Wiener, 2016; Wiener et al., 2012), whereas route repetition relies on egocentric strategies (Waller & Lippa, 2007). We therefore expected performance between the route-retracing and directional-approach tasks to be more strongly correlated than performance between the route-repetition task and the route-retracing or the directional-approach task.

## Materials and method

### Participants

Eighty-one participants were recruited from Bournemouth University (BU) and the surrounding area. These comprised 37 younger participants, 18–32 years of age (23 females, 14 males; mean age: 20.57 ± 3.00), and 44 older participants, 60–82 years of age (29 females, 15 males; mean age: 71.02 ± 5.50). All participants had normal or corrected-to-normal vision. The BU students received credits for their contribution to the project, and the external participants received £12 to cover their expenses. To screen for cognitive impairments, we administered Addenbrooke’s Cognitive Examination III (ACE-III; Mathuranath, Nestor, Berrios, Rakowicz, & Hodges, 2000). All participants scored above the threshold of 82/100 suggestive of cognitive impairments (Hsieh, Schubert, Hoon, Mioshi, & Hodges, 2013; Noone, 2015). The mean ACE-III scores were 92.42 (± 4.79) for the younger participant group and 95.98 (± 2.78) for the older participant group [*t*(53.67) = 3.95, *p* < .001].

### Procedure

Participants came to the Psychology Department at BU for one 90-min test session. They began by completing the ACE-III cognitive assessment to ensure that they scored above the cutoff value (82/100) and were eligible to continue with the study. None of the participants were excluded on this basis. Following this, participants completed the three navigational tasks of the Navigational Test Suite on a computer with a 21-in. screen. We balanced the order in which the navigational tasks were administered between participants and age groups, to ensure that no systematic biases were introduced by potential order effects.

Participants received instructions for each of the navigational tasks explaining what they had to do. Prior to carrying out the actual experimental task they completed a short demo version of the experiment, a substantially shortened version of the task, to ensure that participants understood the navigational tasks. The experimenter answered any questions relating to the protocol of the task, but they did not instruct or encourage participants to use any specific strategy to solve the navigational tasks.

#### The navigational test suite

**Fig. 1:**
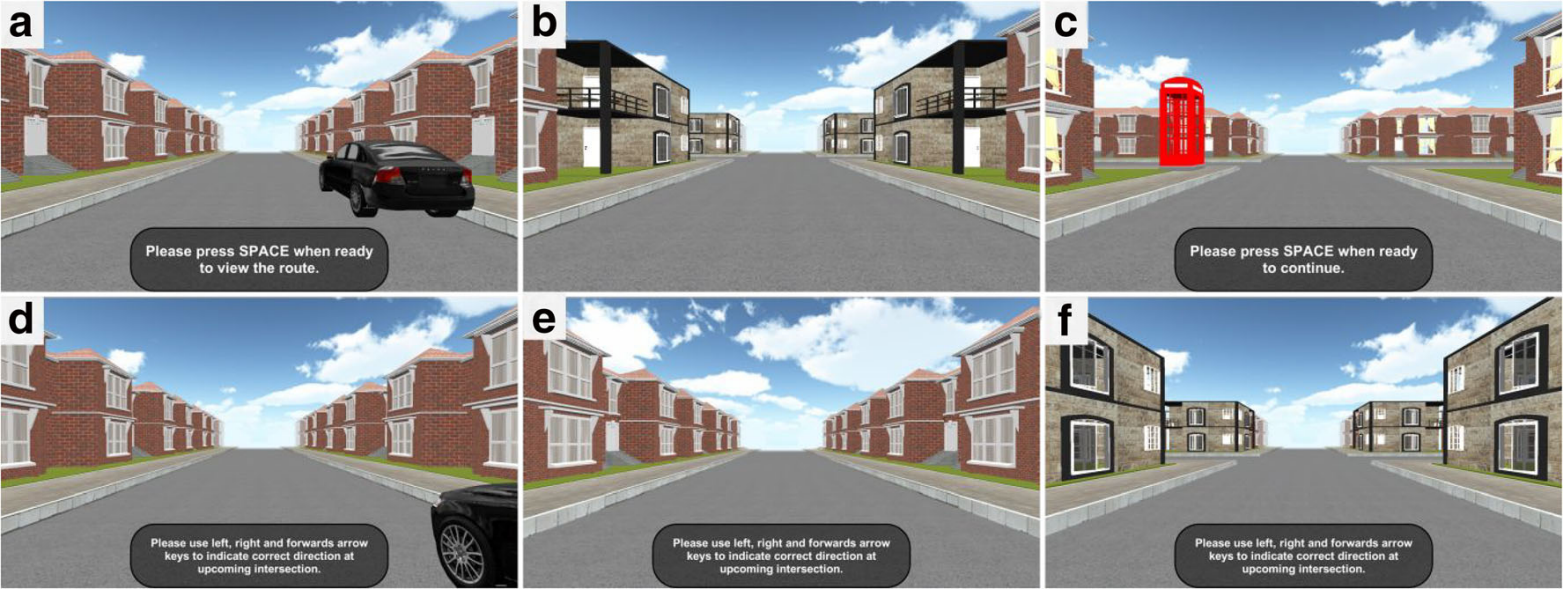
(Top row) Screenshots taken during the learning phase of the route-repetition task. (**a**) Participants “stand” at the starting position and start the passive transportation along a route by pressing the SPACE bar. (**b**) Screenshot taken along the route while approaching an intersection. (**c**) Screenshot taken at the end of the route. Participants initiated the test phase by pressing the SPACE bar. (Bottom row) Screenshots taken during the test phase. (**d**) Participants were transported from the start point toward the first intersection. (**e**) Screenshot taken before the distinctive landmarks at the upcoming intersection were visible. (**f**) Screenshot taken at the end of the navigation phase of a test trial, before the participant has responded

**Fig. 2:**
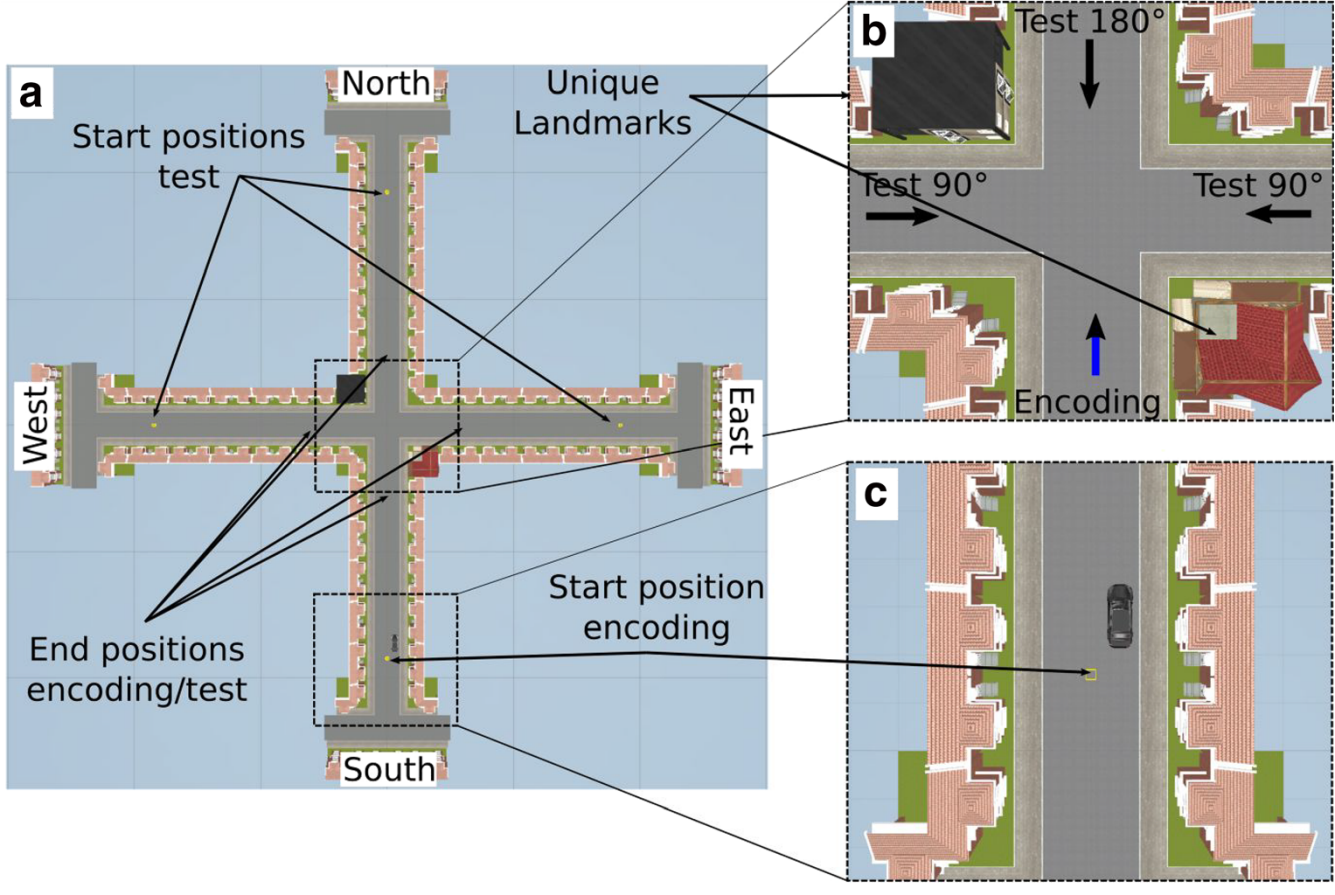
Schematic illustrating the directional-approach task, with an overview of one of the environments used in that task. (**a**) During the encoding phase, participants always approached the intersection with two distinct landmarks positioned at diagonally opposite corners from the street to the south, starting at the black car (**c**). During the test phase, they approached the same intersection from the street to the east, the west, or the north. Note that the car could not be seen when participants were asked to give their response. Critically, the approaches to the intersection from the east and west were misaligned with the encoding situation by 90°, whereas an approach from the north was misaligned with the encoding situation by 180° (**b**)

**Fig. 3:**
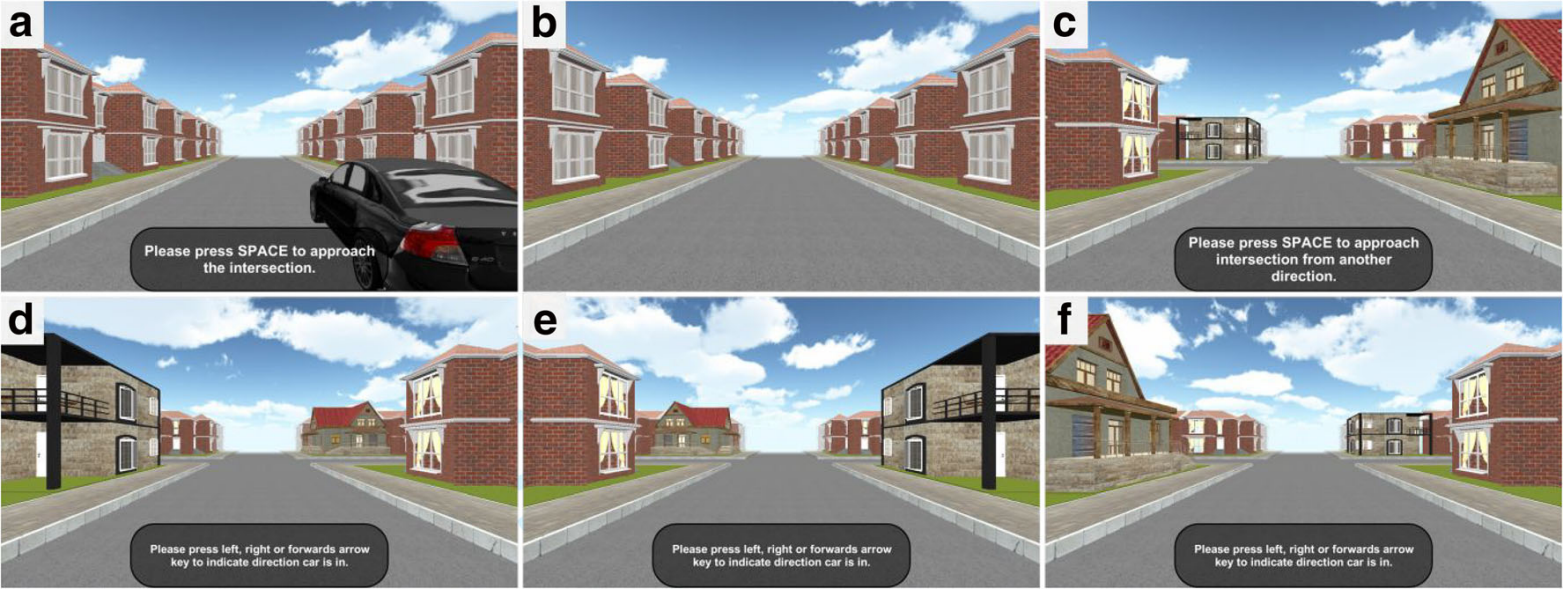
Screenshots taken during the directional-approach task. (Upper row) Encoding phase: (**a**) Participants initiate translation at the beginning of the encoding phase. (**b**) Screenshot taken during passive transportation toward the intersection. (**c**) Final position during the encoding phase. Participants then initiate the test phase. (Lower row) Screenshots taken at the end of the translation in the test phase, when approaching the intersection from the west (**d**), north (**e**), and east (**f**)

On the basis of earlier tasks, we developed a novel navigation test suite that consisted of three navigational tasks: the route-repetition task, the route-retracing task, and the directional-approach task. We used Unity version 5.2.2.f1 (Unity Technologies, Inc. San Francisco, CA, USA) to program the navigation test suite. All tasks made use of the same virtual environment, which consisted of streets and four-way intersections in a residential neighborhood. The houses bordering the streets were all identical (see Figs. 1, 2, and 3 below). The distance between two neighboring intersections was 61 m. In the experiment, participants were passively transported along the route at a speed of 7.6 m/s. We chose this speed, which is faster than a normal walking speed, during pilot testing. At a normal to fast walking speed (between 1 and 3 m/s), passive navigation between two intersections would have required between 20 and 26 s, which both our young and older pilot participants reported as too slow. Given the speed setting for the present experiment, it took 8 s to navigate between two intersections, which was slow enough to give both participant groups enough time to view (and encode) the landmarks along the route.

Depending on the navigational task (explained in more detail below), we introduced unique houses (i.e., distinctive landmarks) to the intersections. The 3-D models used in the VE were either created or we used preexisting models from earlier research projects that were modified to fit in the streets using 3D Studio MAX 2016 (Autodesk). The Navigation Test Suite can be obtained for free from https://osf.io/mx52y/.

Note that we introduced white fog into the environment for all three experimental tasks and in both the learning and test phases. This ensured that participants could only see one intersection at any time. The response times for all tasks were recorded in seconds to the accuracy of five decimal places. However, given a rate of 60 frames per second effectively restricted the temporal resolution to 1/60 of a second.

#### Route-repetition task

The route repetition task is a typical route learning task, designed to assess participants’ ability to learn an unfamiliar route and to repeat this route in the same direction as during learning. (e.g., Waller & Lippa, 2007). It was composed of three experimental sessions, each of which featured a learning phase and a test phase. To assess learning, we presented the same route in each of the three experimental sessions.

At the beginning of the learning phase, participants were positioned in a street next to a black car (see Fig. 1a). After pressing the SPACE key, they were then passively transported along a route that featured six intersections with two left turns, two right turns, and two straight movements. The route stopped at a red phone box (Fig. 1c). Each intersection featured four identical houses (landmarks) positioned at the four corners of the intersection (Fig. 1b). Different intersections featured different houses, such that each intersection could be unambiguously identified. Participants were instructed to learn the route during the learning phase.

The test phase comprised six trials in which participants were asked to reproduce the route from the car to the phone box, one intersection at a time, adding up to a total of six test trials in each experimental session. The first test trial in each test phase started at the car (Fig. 1d), and the other test trials started in the centers of the subsequent intersections, such that the houses at the far end of the current intersection were visible. Participants were then transported passively toward the next intersection along the route (Fig. 1e), were prompted to indicate the direction in which the route continued, and they stopped 20 m before the center of the next intersection (Fig. 1f). Participants could give their response at any time during the test phase, even before passive transportation ended. Immediately after they gave their response, by pressing the corresponding arrow key, they were teleported to the center of the intersection, facing the street that led to the next intersection along the route. In other words, participants did not receive feedback during the test phase. There were no time constraints.

Participants could use two strategies to learn the route, a *sequence-of-direction* strategy (here: left, right, straight, right, left, straight; also referred to as a *route/procedural strategy*; see Zhong & Kozhevnikov, 2016) and/or an *associative-cue* strategy, in which they would associate a direction with a particular landmark (e.g., “Turn right at Blue House”; Waller & Lippa, 2007). The present task was not designed to investigate how these strategies interact during route learning, but the Navigational Test Suite (https://osf.io/mx52y/) does allow presenting the intersections in a random order during the test phase, in which case participants cannot rely on a sequence-of-turns strategy.

#### Route-retracing task

The route-retracing task was designed to assess participants’ ability to find their way back to the starting point after being transported along a route. In earlier work, we argued that such route retracing behavior cannot be explained by simply mirroring egocentric route knowledge when retracing the route. Rather, successfully retracing a route requires allocentric processing (Wiener et al., 2012).

The route-retracing task was identical to the route-repetition task, with the exception that participants in the test phase had to find their way back from the phone box to the black car. In other words, participants had to navigate the route in the opposite direction as compared to the learning phase.

#### Directional-approach task

The directional-approach task was designed to test participants’ ability to encode the configuration of houses (landmarks) relative to the street in which the car was parked. The task is based on a similar task and is thought to assess allocentric processing and perspective taking abilities (de Condappa & Wiener, 2016; Wiener et al., 2013).

The task consisted of 18 independent trials. Each trial began with an encoding phase, in which participants were positioned in a street next to a black car (Figs. 2 and 3a); they were then passively moved toward a single intersection that featured two unique houses (i.e., landmarks) at diagonally opposite corners of the intersection (Fig. 2). Movement stopped 20 m before the center of the intersection (Fig. 3c), such that both unique houses were in view. The participants’ task was to memorize where their car was parked.

In the test phase, participants were passively transported toward the same intersection, but from one of the other three streets (Figs. 2, 3d–f), and participants were asked to indicate the direction in which the car was parked—that is, to indicate the street from which they had approached the intersection during the encoding phase. Movement stopped 20 m before the center of the intersection, but participants could already give their response during the passive transportation. If participants responded before movement stopped, the next trial was initiated immediately.

The car was always parked in the street to the south of the intersection (see Fig. 2). During the test phase, participants approached the intersection from the street to the west, to the north, or to the east (Figs. 3d–f). Importantly, the perspective shift required for aligning the view during the test phase with that experienced during the encoding phase was larger when approaching the intersection from the north (180°) than when approaching from the east (90°) or west (90°; Fig. 2b). Note that participants did not know about these cardinal directions in the experiment—we use them solely for the purpose of presenting the protocol and the data.

There were 18 trials in the directional-approach task, each with a unique combination of houses (landmarks) at the intersection. Six trials required a “left” response, six trials required a “right” response, and six trials required a “straight on” response. The trials were presented in random order. A single trial ended after participants had made their response, and participants did not receive any feedback. Note that, in contrast to the route-repetition and route-retracing tasks, the directional-approach task did not require participants to learn a route with multiple decision points. The directional-approach task therefore had much lower long-term memory involvement than the other tasks.

## Results

### Route repetition and route retracing

#### Performance

**Fig. 4:**
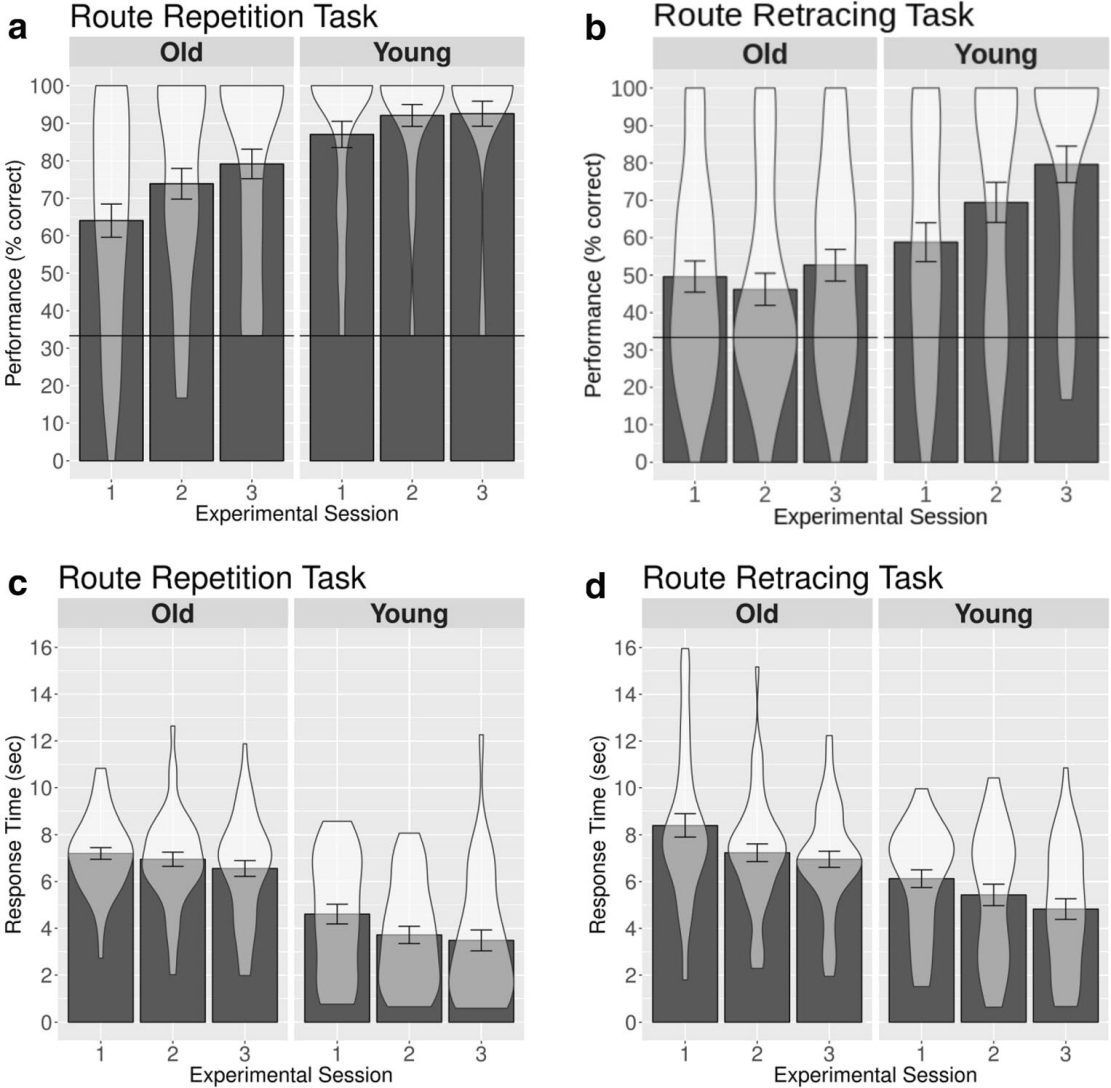
Performance (**a**, **b**) and response times (**c**, **d**) for the older and younger participants in the route-repetition and the route-retracing tasks as a function of experimental sessions. The horizontal lines in panels **a** and **b** represent chance-level performance. The bars represent mean values, the error bars represent standard errors of the means, and we have overlaid the probability density of the participants’ performance or response times at different values. The plots were generated using the ggplot 2 package in R (Wickham, 2016)

We assessed performance as the percentage of trials in which participants provided a correct response. A repeated measures analysis of variance (ANOVA), with the within-subjects factors task (route-repetition task, route-retracing task) and session (1, 2, 3) and the between-subjects factors age group (young, old) and gender (male, female), revealed significant main effects of task [*F*(1, 76) = 53.29, *p* < .001, *η*^2^_G_ = .15],^1^ session [*F*(2, 152) = 10.23, *p* < .001, *η*^2^_G_ = .03], and age group [*F*(1, 76) = 27.99, *p* < .001, *η*^2^_G_ = .11], but not of gender [*F*(1, 76) = 2.54, *p* = .11, *η*^2^_G_ = .01 ]. Specifically, participants performed better in the route-repetition than in the route-retracing task, younger participants performed better than older participants, and performance in the third experimental session was better than that in the first experimental session [*t*(79) = – 4.28, *p* < .01; see Fig. 4].

Only one interaction, between task, session, and age-group, was significant [*F*(2, 152) = 4.85, *p* = .009, *η*^2^_G_ = .01]. To further explore the nature of this three-way interaction, we carried out separate ANOVAs for the route-repetition and route-retracing tasks. We found a significant effect of age group for both tasks [route-repetition task: *F*(1, 78) = 16.53, *p* = .001, *η*^2^_G_ = .12; route-retracing task: *F*(1, 78) = 16.09, *p* < .001, *η*^2^_G_ = .10], demonstrating that the younger group performed better than the older group in both tasks. In both tasks there was an effect of session [route-repetition task: *F*(2, 156) = 8.44, *p* < .001, *η*^2^_G_ = .03; route-retracing task: *F*(2, 156) = 4.65, *p* = .011, *η*^2^_G_ = .03], with better performance in later experimental sessions. In the route-repetition task, the interaction between age group and experimental session was not significant [*F*(2, 156) = 1.55, *p* = .216, *η*^2^_G_ = .006]. In the route-retracing task, by contrast, the interaction between age group and experimental session was significant [*F*(2, 156) = 3.09, *p* = .048, *η*^2^_G_ = .02]. This interaction was driven by a significant increase in performance in the young participant group between Sessions 1 and 3 [*t*(69.72) = – 2.92, *p* = .005], whereas the older age group did not significantly improve their performance over the course of the experiment [Session 1 vs. Session 3: *t*(85.99) = – 0.51, *p* = .608]. These additional analyses suggest that the significant three-way interaction between age group, task, and session in the original ANOVA was driven by a significant learning effect in the young participant group in the route-retracing task, which was absent in the older participant group (see Fig. 4).

#### Response times

Response time represents the time between the onset of motion in the test phase until participants responded.

A repeated measures ANOVA with the between-subjects factors age group (young, old) and gender (male, female) and the within-subjects factors task (repetition, retrace) and experimental session (1, 2, 3) revealed significant main effects of age group [*F*(1, 76) = 31.70, *p* < .001, *η*^2^_G_ = .21], task [*F*(1, 76) = 20.44, *p* < .001, *η*^2^_G_ = .04], and experimental session [*F*(2, 152) = 39.16, *p* < .001, *η*^2^_G_ = .04], but no effect of gender [*F*(1, 76) = 2.00, *p* = .161, *η*^2^_G_ = .017]. Response times were longer in the old than in the young age group, longer for the retrace than for the repetition task, and decreased over the experimental sessions. None of the interactions were significant.

#### Directional-approach task

**Fig. 5:**
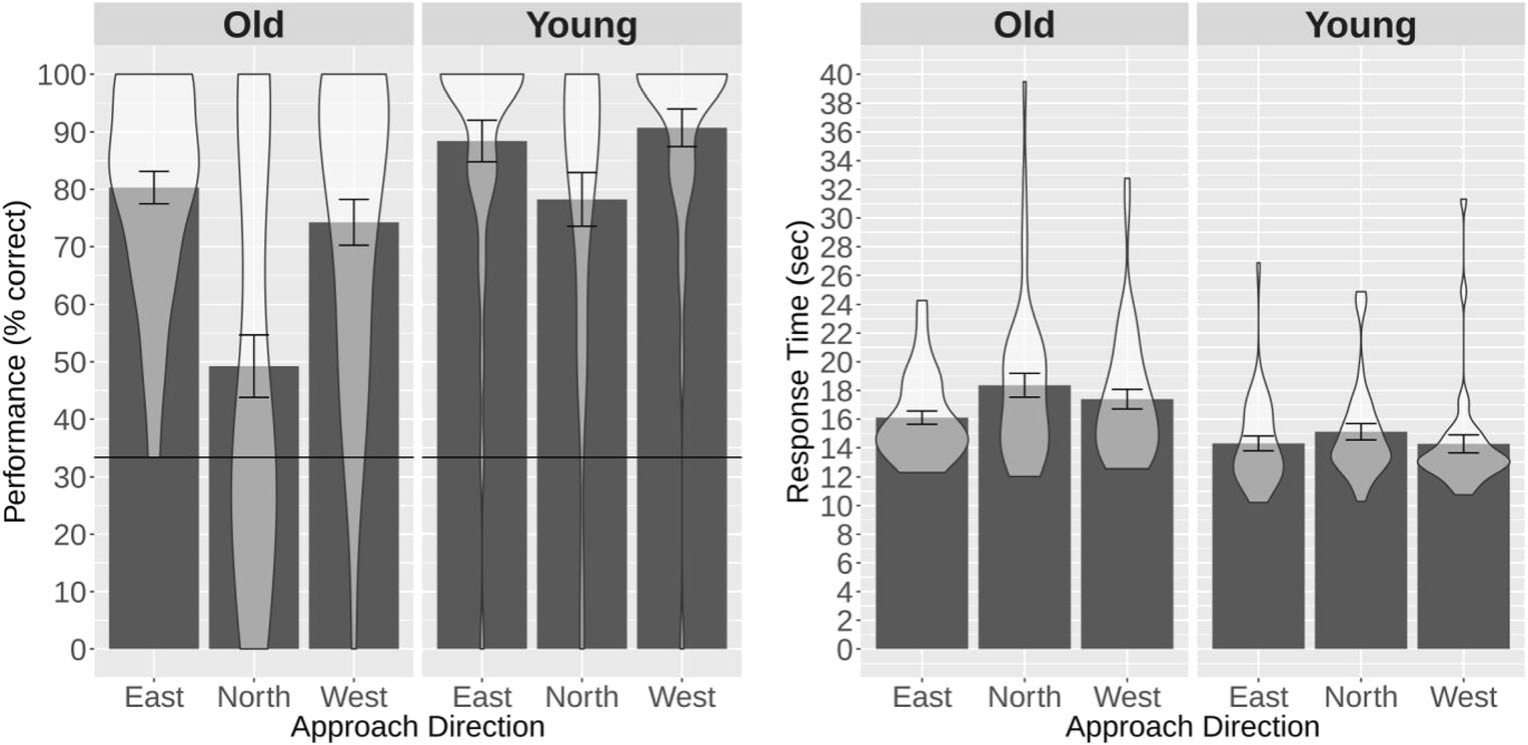
Performance (left) and response times (right) for the older and younger participant groups in the directional-approach task. The horizontal line in the left plot represents chance level. Note that approaching the intersection from the east or west was misaligned with the encoding view by 90°, whereas approaching the intersection from the north was misaligned with the encoding view by 180°. Bars represent mean values, error bars represent standard errors of the means, and we have overlaid the probability density of participants’ performance or response times at different values. The plots were generated using the ggplot 2 package in R (Wickham, 2016)

A repeated measures ANOVA with the within-subjects factor approach direction (west, north, east) and the between-subjects factors age group (young, old) and gender (male, female) revealed significant main effects of approach direction [*F*(2, 152) = 28.13, *p* < .001, *η*^2^_G_ = .13] and age group [*F*(1, 76) = 15.21, *p* < .001, *η*^2^_G_ = .11], but no effect of gender [*F*(1, 76) = 3.21, *p* = .076, *η*^2^_G_ = .025]. Specifically, younger participants performed better than older participants, and performance was significantly worse for an approach from the north than for an approach from either the east [*t*(79) = 5.88, *p* < .001] or the west [*t*(79) = 5.46, *p* < .001] (see Fig. 5). Performance for the west and east approaches was similar (*p* = .332). Only the interaction between age group and approach direction rendered a significant result [*F*(2, 152) = 5.29, *p* < .005, *η*^2^_G_ = .03]. This interaction was driven by a stronger decline in performance in the older group than in the younger group when approaching the intersection from the north rather than from the east or west [*t*(76.74) = – 2.53, *p* = .013].

To investigate whether performance increased over the course of the 18 trials, we calculated correlations between performance and trial number (1–18). We did not find significant correlations when we combined the young and old participants (*r* = .36, *p* = .146) or when analyzing the age groups independently (young: *r* = .39, *p* = .110; old: *r* = – .25, *p* = .319). This suggests that participants’ performance did not improve significantly over trials.

#### Response time

A repeated measures ANOVA with the within-subjects factor approach direction (west, north, south) and the between-subjects factors age group (young and old) and gender (male, female) revealed only a significant main effect of age group [*F*(1, 76) = 11.97, *p* < .001, *η*^2^_G_ = .10], and did not reveal any significant interactions (see Fig. 5). Specifically, response times were longer for the old than for the young age group.

### Correlations between navigational tasks

Because all participants completed all three navigational tasks (directional-approach, route retracing, and route repetition), we were able to correlate performance between the different tasks. We predicted that we would observe a significant correlation between the route-retracing and directional-approach tasks, both of which have been suggested to require some allocentric processing (Wiener et al., 2013; Wiener et al., 2012). Since the route-repetition task was the only one that could be solved using a purely egocentric strategy, we did not expect significant correlations between the route-repetition task and either the directional-approach task or the route-retracing task. To control for differences in age, which above was shown to affect performance, we calculated correlations in which we partialed out chronological age. The results of these partial correlations are summarized in Table 1. As predicted, we found a significant correlation between the directional-approach task and the route-retracing task. In contrast, the correlations between the route-repetition task and both the directional-approach and route-retracing tasks were not significant (or did not survive adjustment for multiple comparisons).

**Table 1:** Results of the partial correlations between the three navigational tasks

Route-repetition task vs. Route-retracing task	*r*(77) = .15, *p* = .20
Route-repetition task vs. Directional-approach task	*r*(77) = .23, *p* = .04
Route-retracing task vs. Directional-approach task	*r*(77) = .32, *p* = .004

To control for multiple comparisons, we used a Bonferroni-adjusted alpha level of .0167. Note that direct comparisons between the three correlation coefficients using Steiger’s *Z* test did not reveal significant differences (all *p*s > .05, with *z* scores ranging from 0.61 to 1.26)

## Discussion

The main aim of the present study was to introduce and evaluate three new navigational tasks. On the basis of earlier published work, we present tasks addressing the ability to learn a route, the ability to retrace a recently travelled route, and the ability to learn and use a configuration of landmarks. When designing these tasks, we aimed to make them ecologically valid, and easy to explain and administer, such that they would be suitable for use with older adults and patient groups in the future.

The design of the route-repetition task was based on earlier research (e.g., Head & Isom, 2010). In each of three experimental sessions, participants were passively navigated along a route featuring six intersections starting from a parked car and ending at a phone box. In the test phase, participants were navigated toward each intersection in the same order as during learning and were asked to indicate the direction in which the route continued. We were able to replicate earlier findings (Head & Isom, 2010). Specifically, whereas both our young and our older participant group showed learning over the course of the experiment, the young participant group performed better than the old participant group. Analyses of response times, which showed longer response times in the older age group (Salthouse, 1996), suggest that the age-related decline in route-learning abilities cannot be explained by a speed–accuracy trade-off.

In the present route-learning task, participants can use two egocentric strategies to solve the task, an associative-cue strategy, in which they associate landmarks with movement directions, and a procedural or sequence-of-turns strategy, in which they remember the sequence of turns required to navigate the route (Waller & Lippa, 2007). Although it is beyond the scope of this study to investigate the relationship between these strategies, it is important to point out that both could be affected by aging. First, the associative-cue strategy relies on associative learning, which is affected by cognitive aging (Naveh-Benjamin, Hussain, Guez, & Bar-On, 2003). Second, the learning of sequences of turns along a route relies on the hippocampal circuit (Iglói, Doeller, Berthoz, Rondi-Reig, & Burgess, 2010), which undergoes substantial functional and structural changes already during the typical aging process (Fjell, McEvoy, Holland, Dale, & Walhovd, 2013). Current research in our laboratory aims to disentangle the contributions of the associative-cue and the sequence-of-turns strategies and the effect that cognitive aging has on each strategy.

The route-retracing task was identical to the route-repetition task discussed above, but it required that participants navigate from the end of the route back to the start location. This is a frequent real-world navigational task that has received very little attention in the literature. The task used here was inspired by earlier work (Wiener et al., 2012), and whereas the exact procedure was slightly different from the one we used earlier, we were able replicate the main findings: First, our young participants outperformed our older participants; second, we found that our young, but not our old, participant group improved over the course of the experiment.

It is important to note that egocentric strategies, such as the associative-cue strategy (“Turn left at church”) or the sequence-of-turns strategy discussed above, do not directly support route retracing, in that intersections are approached from a different direction than during the encoding of the route. Although it is conceivable that participants would simply mirror the direction of the turn required at each intersection when retracing a route, this does not explain why our older age group did not show any learning over the experimental sessions. Moreover, if route repetition and route retracing relied on the same cognitive strategies, we would expect to find a significant correlation between performance in these tasks, which we did not find. In contrast, we found a significant correlation between the route-retracing and directional-approach tasks (discussed in more detail below). This is in line with the explanation that route retracing requires abstracting from a purely egocentric representation that can be achieved by encoding the spatial relationship between the street from which an intersection was approached and the street on which the route proceeded (Wiener et al., 2012). The encoding and processing of such allocentric representations are affected by cognitive aging (Harris & Wolbers, 2012; Moffat, Kennedy, Rodrigue, & Raz, 2007; Moffat & Resnick, 2002), possibly resulting from age-related hippocampal neurodegeneration (Raz et al., 2005).

The directional-approach task required that participants encode the spatial relationship of landmarks at an intersection and the direction or street from which this four-way intersection was approached. In the test phase, participants then approached the same intersection from any of the remaining three directions/streets. The protocol was based on earlier work (Wiener et al., 2013) that had shown age-related declines in participants’ ability to channel into a route when approaching an intersection from a different direction than during learning. In contrast to the earlier protocol, participants were not required to learn a route in the present study, but only the direction from which they had approached a single intersection during learning. Despite these methodological differences, we found an age-related decline in participants’ ability to solve the task. Importantly, we found an interaction between approach direction and age group, with older adults showing stronger performance decrements when the approach direction in the test phase was misaligned with the encoding situation by 180° instead of 90°.

These findings corroborate earlier notions that the task requires perspective shifts in order to align the current viewpoint with the encoded viewpoint (de Condappa & Wiener, 2016), a process that is affected by cognitive aging (Watanabe, 2011). In line with this interpretation, response times were longer when the direction in the test phase was misaligned by 180° than when it was misaligned by 90°. However, this difference was not statistically significant. Hence, this issue needs to be addressed in future studies, potentially by introducing an additional test situation in which the viewpoint during test is aligned with that during encoding (0° misalignment). Finally, we did not find a significant correlation between the trial number and performance in the directional-approach task, which suggests that this task did not benefit from or require training.

As we stated in the Procedure section, participants were neither instructed nor encouraged to use any specific strategy to solve the navigational tasks. It is therefore possible that the reported age-related differences resulted, at least partly, from differences in strategy selection or preferences between age groups (e.g., Wiener et al., 2013; Zhong et al., 2017). Future studies should therefore consider addressing the impact of strategy selection or differential use of reference frames (egocentric vs. allocentric) to encode spatial information on task performance by different age groups, potentially by explicitly instructing participants to employ different strategies to solve the task.

We have introduced here a new experimental software package for three navigational tasks that are adapted from earlier protocols (Head & Isom, 2010; Wiener et al., 2013; Wiener et al., 2012). The new navigational tasks presented here were carefully designed such that they (i) resemble real-world navigation situations more closely than the original tasks, (ii) are easy to explain to participant groups who may have little experience with virtual environment technology, and (iii) are easy to modify by editing configuration files to address other research questions or to test other participant groups. For example, our young participant group showed very strong performance in the route-repetition task. To further study route learning in young participants, it would be easy to lengthen the route or, to address the interaction of the associative-cue and sequence-of-turns strategies (Waller & Lippa, 2007), to present the intersection in random order during the test phase. Despite adapting the protocols, we replicated the original main findings, which demonstrates that the new navigation test suite introduced here has similar task demands. By making the test suite freely available to other research groups (https://osf.io/mx52y/), we hope to contribute to the development of normative data sets that will be crucial for the development of navigational assessments in a clinical context.

## Footnotes

1 Note that we report generalized eta-squared rather than partial eta-squared, because it provides comparability across between- and within-subjects designs (Bakeman, 2005).
